# A Wireless Real-Time Continuous Monitoring System for the Internal Movements of Mountain Glaciers Using Sensor Networks

**DOI:** 10.3390/s22239061

**Published:** 2022-11-22

**Authors:** Shimeng Wang, Aihong Xie, Jiangping Zhu

**Affiliations:** 1 State Key Laboratory of Cryospheric Sciences, Northwest Institute of Eco-Environment and Resources, Chinese Academy of Sciences, Lanzhou 730000, China; 2 University of Chinese Academy of Sciences, Beijing 100049, China

**Keywords:** glacier internal movement, wireless monitoring, real-time monitoring, glacier disasters warning

## Abstract

With the escalation of global warming, the shrinkage of mountain glaciers has accelerated globally, the water volume from glaciers has changed, and relative disasters have increased in intensity and frequency (for example, ice avalanches, surging glaciers, and glacial lake outburst floods). However, the wireless monitoring of glacial movements cannot currently achieve omnidirectional, high-precision, real-time results, since there are some technical bottlenecks. Based on wireless networks and sensor application technologies, this study designed a wireless monitoring system for measuring the internal parameters of mountain glaciers, such as temperature, pressure, humidity, and power voltage, and for wirelessly transmitting real-time measurement data. The system consists of two parts, with a glacier internal monitoring unit as one part and a glacier surface base station as the second part. The former wirelessly transmits the monitoring data to the latter, and the latter processes the received data and then uploads the data to a cloud data platform via 4G or satellite signals. The wireless system can avoid cable constraints and transmission failures due to breaking cables. The system can provide more accurate field-monitoring data for simulating glacier movements and further offers an early warning system for glacial disasters.

## 1. Introduction

In recent decades, global warming has unequivocally been accelerating [1], especially with respect to the Earth’s three poles. The Third Pole, which largely consists of the Tibetan Plateau (TP hereafter), has undergone significant warming, at a rate that is almost twice as fast as the global mean in the past decades [2]. The Arctic Pole is warming at a doubled rate, and this rate is even three times or nearly four times faster than the global rate since 1979 [3,4,5]. The polar amplification phenomenon has also existed on the Antarctic surface in recent decades [6]. Polar amplification can accelerate the shrinking of glaciers [7,8], since most glaciers exist at the Earth’s Third Pole and other mountain regions. The current shrinkage provides direct and unequivocal effects on water resources, and relative disasters occur with increasing intensity and frequency, such as ice avalanches, surging glaciers, and glacial lake outburst floods (GLOFs) [9,10,11,12,13]. However, how can we predict future glacial ice volumes and any related water resource changes? The response of glaciers relative to climate change is very complex and occurs via the transmission of dynamic waves, which is the main factor that restricts the advance and retreat of glaciers and whether the transmission of dynamic waves can reach the end or bottom of glaciers [14,15]. Based on glacial dynamic processes, glacial response models can elucidate the impact of glacial changes on hydrology and determine the time-lag relationship of glacial responses relative to climate change, thus identifying the quantitative relationship between climate, glaciers, and hydrology [16].

A glacier is a large accumulation of ice and snow, which is a product of a certain climate and terrain, and slowly moves over land. Under the influence of gravity fields, the movement speed at different depths of the glacier is different [17,18,19]. As a result, the instruments and equipment using communication cables cannot function in glaciers for a long period of time [20]. Therefore, wirelessly monitoring the internal dynamic parameters has become necessary, especially at the Third Pole, which is a typical area with cryosphere hazards [21]. Most previous research studies focused on the glacier’s surface, and the present monitoring methods mainly depend on traditional methods, such as material balance observation stakes and snow pits [22,23], artificial tunnels [24,25,26,27], ice hole recorders [28,29,30], negative thermal resistors [31], and satellite observations [32,33,34].

However, because of the relatively thin thickness (usually less than 200 m), fast movement speed, serious ice hole shrinkage, and high maintenance cost of the instruments in the later stage of mountain glaciers, little attention has been paid to monitoring the internal real-time movement of mountain glaciers, with the omnidirectional and high-precision monitoring being a technical bottleneck. In fact, there is a long history of the use of wireless radio frequency propagation through ice since the 1960s, using radio-echo sounding, radar, and other technologies to detect a variety of physical glacial characteristics. Theoretically, the wavelength of ultra-high frequencies (UHF) (300 MHz to 3 GHz) is generally longer than the englacial water body with respect to its transmission path, so it can pass through smoothly. However, glacier ice is a heterogeneous and lossy medium. When the wavelength passes through, its transmission distance will be limited by scattering and attenuation loss (its attenuation coefficient is 0.01 dB m^−1^). Therefore, to a large extent, the transmission distance is determined by the power of the transmitting module [35,36]. Therefore, this paper will design a wireless real-time monitoring system to measure the internal movement parameters of mountain glaciers using sensor networks. This design focuses on the bottom temperature, pressure, humidity, and power supply voltage parameters. When the technology is mature and reliable, this wireless real-time monitoring system will expand its functions and can increase the types of monitoring parameters. Its measurement parameters can be used for comprehensive monitoring, prediction, and early warning of glacial disasters. Based on the principle of digital communications [37], the system integrates sensor technologies, wireless communication technology, and network information technology, and it analyzes the working principle and structure of wireless monitoring systems [38], including their basic functions, workflow, preliminary test results, and future work plans.

The fundamental structures of the monitoring system, as well as parameters of the corresponding sensors, are summarized in Section 2. In Section 3, the system’s working principles are reviewed. Section 4 compares and contrasts the monitoring result from the system’s performance, including its power consumption and preliminary tests. Section 5 concisely summarizes the system and provides a way forward.

## 2. System Structures

**Figure 1:**
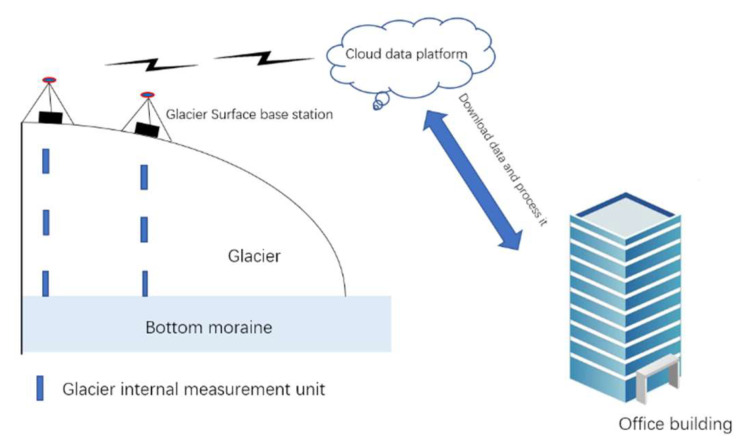
Schematic depiction of monitoring a glacier’s internal parameters with the wireless real-time system.

The monitoring system consists of a glacier internal measurement unit and glacier surface base station (Figure 1). Arduino Nano is the core module of the glacier internal measurement unit, and Arduino Mega2560 is employed for the glacier surface base station. We chose the Arduino series module, since it is an open-source platform with numerous users and a wide range of applications. Most importantly, for non-computer-professional researchers, it does not require consideration of the underlying code, and the supporting integrated development environment (IDE) has a friendly interface.

The glacier internal measurement unit periodically measures the monitoring parameters, and then it sends the data to the glacier surface base station via the wireless transmission module. The glacier surface base station stores the data in the local secure digital memory (SD) card, and then it uploads the received data to the cloud data platform through 4G communication or satellite signals. After logging into the cloud data platform account, we can download the uploaded data anytime and anywhere; the working status of the system can then be viewed.

### 2.1. Glacier Internal Measurement Unit

**Figure 2:**
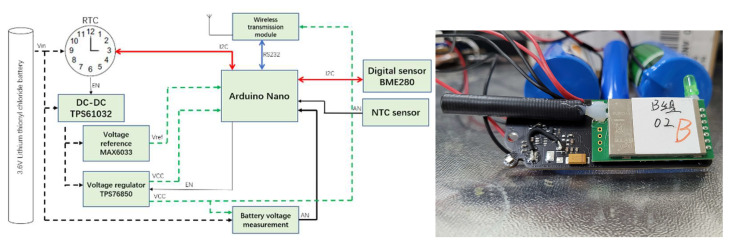
Structure diagram (**left**) and an example of the real sensors (**right**) for the glacier internal measurement unit.

As shown in Figure 2, the glacier internal measurement unit consists of a data processing section, a power supply section, an RTC clock, sensors, and a wireless transmission module. The core processing unit is an Arduino Nano, and its microcontroller unit (MCU) is ATmega328, with a working voltage of 1.8 V–5.5 V, flash memory of 32 Kb, EEPROM of 1 Kb, 8 analog input ports, 14 digital ports, an operating frequency of 16 MHz, a working current of about 0.2 mA, a power-off mode of 0.1 µA, and operating temperature of −40 °C to 85 °C [39].

The RTC clock module comprises a DS3231 with a low-power (minimum power consumption 3µA), high-precision I2C real-time clock device and an integrated temperature-compensated oscillator, to ensure the accuracy of the device (operating environment −40 °C~+85 °C, annual drift ±3.5 ppm) [40].

We selected Lora E32 as the wireless transmission module, and its RF chip is SX1278 from SEMTECH with supporting LoRaTM Direct Sequence Spread Spectrum technology, which can provide longer communication distances, data encryption, and compression. The output is 30 dBm and it works in the 410 Mhz~441 MHz range with industrial-grade standard designs. It can support an environment at −40 °C~+85 °C over a long period of time, and the worldwide license-free ISM 433 MHz band is supported [41].

The power supply uses disposable lithium thionyl chloride batteries of capacity type ER34615, an open circuit voltage of 3.6 V, and termination voltage of 2.0 V. This battery has a high specific energy (590 wh/kg), excellent high and low temperature performances (−55 °C~+85 °C), low self-discharge characteristics (1%), and an ultra-long standby lifetime (10 years). The instantaneous pulse discharge current can reach 300 mA, and the nominal capacity is 19000 mAh [42].

The temperature sensor is a negative temperature coefficient (NTC) thermistor, which was made by the State Key Laboratory of Frozen Soil Engineering (SKLFSE), Northwest Institute of Eco-Environment and Resources, Chinese Academy of Sciences. The model is SKLFSE-TS, and the accuracy of the temperature measurement is better than 0.05 °C [43].

For the system we chose the integrated environment sensor BME280, which has a low power consumption (3.6 µA @1 Hz), to mainly measure pressure. The test range of the digital sensor is 300 hPa~1100 hPa, the root mean square noise of the pressure is 0.2 Pa (about 1.7 cm), and the sensitivity error is ±0.25%. If it was possible to measure humidity in the sealed container, the tolerance of the relative humidity was ±3%, the input voltage was 1.2 V~3.6 V, and the operating temperature was −40 °C~+85 °C [44].

### 2.2. Glacier Surface Base Station

**Figure 3:**
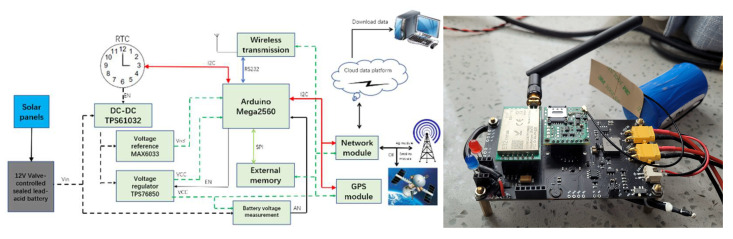
Structure diagram (**left**) and an example of the real sensors (**right**) for the glacier surface base station.

As shown in Figure 3, Arduino Mega2560 is the processing module, and ATMega2560 is the microcontroller unit (MCU). The glacier surface base station unit has an advanced RISC architecture and can execute 135 instructions in a clock cycle, with 32*8 universal working registers, a 256 Kb system with self-programmed flash memory, and low-power designs, and it can support multiple working modes while idle; the input voltage was 4.5 V~5.5 V, and included many differences between the working (4 MHz, Vcc = 3 V, 5 mA) and power-off (disable WDT, Vcc = 3 V, <1 µA) modes. The operating temperature can range from −55 °C to +125 °C [45].

The RTC module and wireless transmission module are the same as that in the glacier internal measurement unit. The power supply uses a combined mode comprising 12 V valve-controlled sealed lead acid batteries and solar panels. We chose this type of battery for the following advantages: a long service life (more than 10 years), very low self-discharge rate (less than 2% per month at 25 °C, about 1/5–1/4 of other batteries), wide temperature range from −15 °C to +45 °C, good sealing, and safe and reliable explosion-proof exhaust system. The solar panels store the generated energy in batteries and then supply power to the glacier surface base station unit. This type of energy supplement can avoid shortening the life of the batteries due to being under-voltage. The solar controller can monitor the voltage parameters of the batteries in real time. When the set value is reached, the charging stops, as overcharging will also shorten the life of the batteries.

The network module employed the A7670C 4G module to support the three major domestic operators, 4G full band, power supply at 3.4 V~4.2 V, sleep power consumption at <3 mA, and operating temperatures from −30 °C~+80 °C [46].

When the system is in glacial regions where there is no 4G signal, we selected the satellite communication module instead of the 4G module to upload the data. Since the global position system (GPS) module can be used for field deployments to locate the glacier surface base station, the system deploys several GPS modules at the same time, and GPS data are used to analyze the moving speed of the glacier surface base station.

## 3. System Working Principles

**Figure 4:**
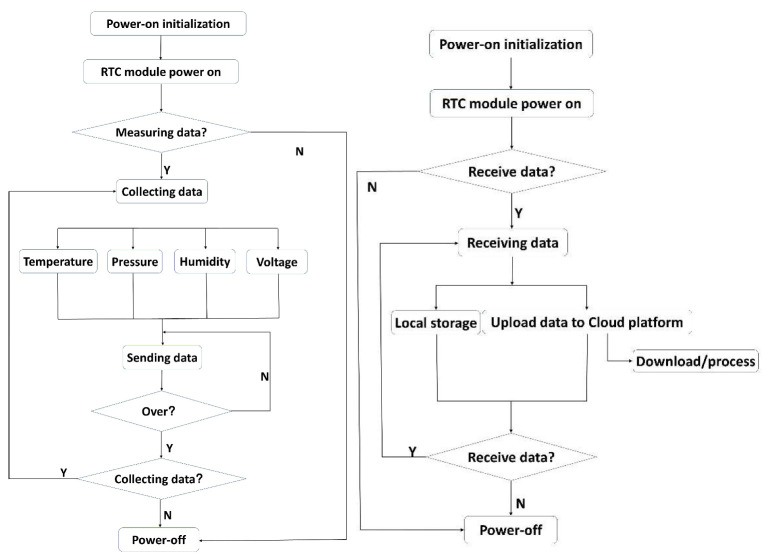
System workflow diagram for glacier internal unit (**left**) and surface base station (**right**).

The system’s workflow consists of two parts (Figure 4): the glacier internal measurement unit and the glacier surface base station. The core module for the glacier internal measurement unit is Arduino Nano, which is powered off during rest times to minimize the energy consumption, except when the Nano and the accessory circuit are collecting/sending data. The RTC module acts as a watchdog. In addition to providing a time baseline for the system, RTC also powers on the Nano system and its accessory circuits at a set time. After Nano completes the data collection and sends the data according to the set program, the Nano and accessory circuit are powered off again, and they await the next power-on period to repeat the same action.

Arduino Mega2560 is the core module of the glacier surface base station. The energy-saving control circuit is consistent with the glacier internal measurement unit, and the local storage and data uploading to the cloud platform are added to this part.

When powered on and operating, the system first judges whether it is the time to receive data. If so, the set procedure is to follow the next step. Otherwise, it will enter the high-efficiency to energy-saving state. Mega2560 and its accessory circuit are powered off, and the RTC module is the only module kept on duty; electrode consumption is low at ~3 µA. When the set alarm clock arrives, the SQW pin of the RTC module will be at a high level, and the main control circuit starts. Mega2560 and its accessory circuit are powered on, and they operate according to the set procedure after initialization.

To ensure the integrity of the sending/receiving data, two measures are taken. Firstly, the power-on time of the glacier surface base station is slightly earlier than that of the glacier internal measuring unit, and the power-off time comes after the glacier internal measuring unit. The high stability of DS3231 (RTC module) can ensure the clock’s consistency between the two parts. Secondly, the glacier surface base station will reply “ok” to the glacier internal measurement unit after receiving the qualified data, and the latter will send the next group’s data after receiving the “ok” message. If the glacier internal measurement unit does not receive the “ok” message within 5 s, it will resend it once, and there are only two resend opportunities to save power consumption.

**Figure 5:**
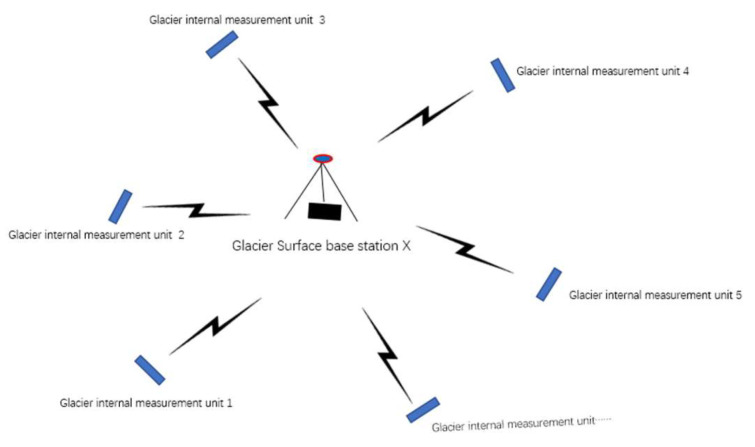
Schematic depiction of the star topology for a system.

Figure 5 shows the star topology used to send data between the glacier internal measurement unit and the glacier surface base stations. The star topology has the following advantages: a simple structure, simple access protocol, easy network monitoring and management, and high reliability. Fault diagnoses and isolation are easy, and the fault of a single glacier internal measurement unit only affects itself and does not affect the entire network. As for convenient services, glacier surface base stations can easily provide services and reconfigure the network for each site. Unfortunately, the glacier surface base station is heavily burdened, forming a “bottleneck”. Once a failure occurs, the entire network will be affected.

A system consists of one glacier surface base station and several glacier internal measurement units. To acquire more persuasive data, we deployed several sets of systems on one glacier, which form one monitoring sensor network. In order to distinguish the measurement data of each unit from each system, we assigned unique IDs and the core processing Arduino module for each unit. Via the corresponding identified IDs, glacier internal measurement units can send the data to the corresponding glacier surface base station; that is, the glacier internal measurement units and their corresponding glacier surface base station are bounded one by one. Once bounded, unless deleted artificially, the system will not be unbounded automatically.

In addition to setting the alarm clock for power on/off function, the RTC module also provides a reference time for the system. Measurements are stored in the same file in chronological order for scientific researchers to retrieve and use.

The wireless real-time monitoring system has two working modes, automatic and manual, and the former is the main working mode. When it is in the automatic working mode, it has two states: active state and power-off state. The switching between the two states is controlled by the RTC module. When the set time for measuring the data arrives, the SQW pin of the RTC module outputs a high level to the EN pin of the power management chip to supply power to the entire system. When the system enters the active state, it will complete the system’s self-test, data measurement, sending/receiving data, and other related work according to the set program; then, the SQW pin of the RTC module outputs low levels to the EN pin of the power management chip, powering off the entire system, and the system enters the powered-off state. This state is its main state. In order to save power consumption, this state is particularly important, especially for glacier internal units; since its power supply is a disposable battery. With the limited energy, the lower the power consumption, the longer the system runs. The RTC module also enters the sleep state, leaving only the clock working, and it waits for the system’s next activation. The glacier internal measurement unit generally takes less than 2 s to complete all setup procedures, and the main power consumption (maximum) occurs at the time of sending data. After completing the established procedures, the SQW pin of the RTC module turns to low levels, and the system is powered off and enters the ultra-low power consumption stage until the time to measure the data comes again.

The manual mode is mainly used to check and evaluate the operating state of the system, and it has specific commands to complete a running state of the system. After completing the command, if the operation of sending/receiving data does not occur, the system immediately enters a sleep state to save power consumption.

## 4. System Performances

### 4.1. Power Consumption

**Figure 6:**
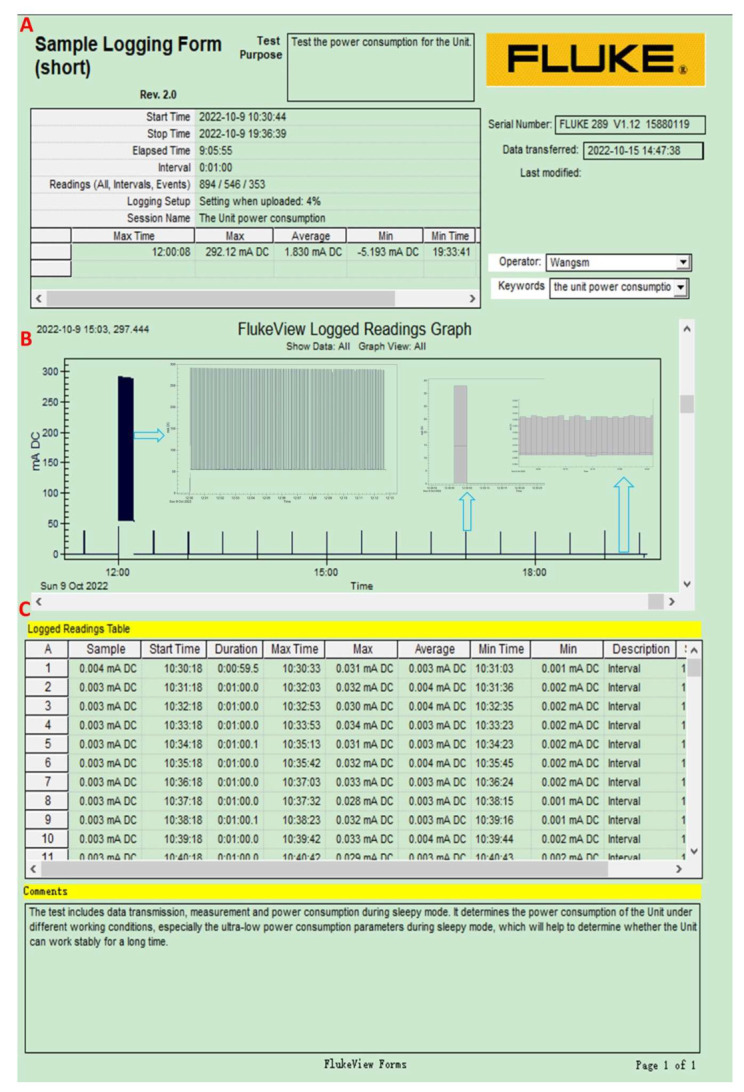
Power consumption measurement for the glacier internal measurement unit, including the logging descriptions (**A**, upper panel), FlukeView GRAPH (**B**, middle panel), and the detailed data (**C**, bottom panel). The black wide thick bar, the black thin bars, and the horizontal line between the two black thin bars refer to the power consumption when the unit sending measurement data within a day at a set time (12:00 a.m.), the measuring data, and when it functions in the sleep mode, respectively. Details are shown in the inner corresponding panel.

The glacier internal measurement unit is powered by DC 3.6 V (Table 1), and the sampling interval is 30 min (Figure 6). The maximum (average) power consumption is 0.894 W (sending phase, lasting about 34.5 s/one day). The power consumption is 0.054 W (lasting about 3.6 s/one time), 0.0005 W (lasting about 34 s/one time), and 0.00001 W (3 μA) for the active mode, storage mode, and sleep mode, respectively.

**Figure 7:**
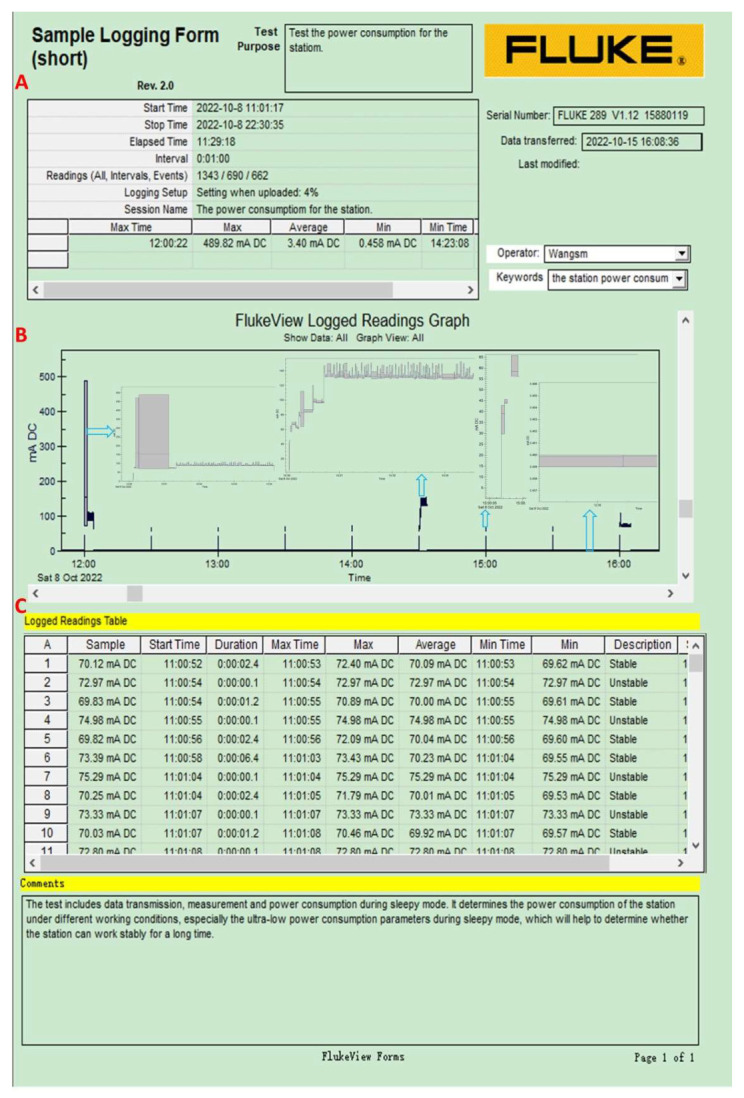
Power consumption measurement for the glacier surface base station, including the logging descriptions (**A**, upper panel), FlukeView GRAPH (**B**, middle panel), and the detailed data (**C**, bottom panel). The black long bar on the left refers to the power consumption when the station is receiving data from the corresponding unit and uploading the data to the cloud data platform at a set time (12:00 a.m. in the figure). The black short thin bar refers to the surface station’s (itself) power consumption when measuring and uploading the data to the cloud data platform at a set time (14:30 p.m.). The black discontinuous lines refer to power consumption when measuring data. The horizontal line between the two black short thin bars is the power consumption of the station in the sleep mode. The details are shown in the inner corresponding panel.

The glacier surface base station is powered by DC 12 V (Table 1), and the sampling interval is also 30 min (Figure 7). The maximum power consumption is 4.12 W (uploading phase, lasting about 48.9 s/one day, one unit). The power consumption is 4.04 W (lasting about 7.4 s/a day, one unit), 0.85 W (lasting about 1.2 s/one time), 0.65 W (lasting about 0.1 s/one time), and 0.006 W (0.046 mA) when saving one unit’s data in the active mode, storage mode, and sleep mode, respectively. The total power consumption of a base station is determined by its related units: the more units there are, the higher the power consumption.

### 4.2. Preliminary System Test Results

**Figure 8:**
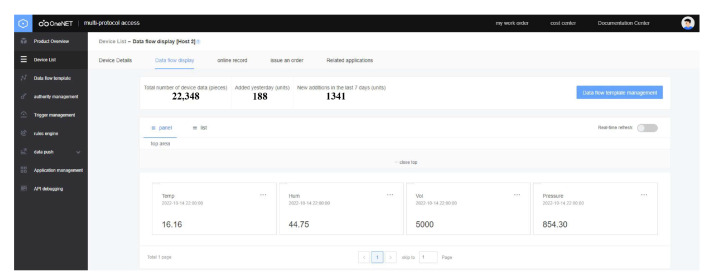
Cloud data platform.

Up until now, the system has been in operation for more than 2 years from September 2020. The test results show that the design of this version runs stably with a packet loss of 3.7‰, which meets the relevant specifications of wireless communication technologies. The glacier internal measurement unit sends data to the glacier surface base station every half an hour. After receiving the data, the latter stores the data in the local storage card and then uploads the received data to an account opened by the cloud data platform (Figure 8). Users can log on to the account anytime and anywhere to check the operation of the system and download data.

**Figure 9:**
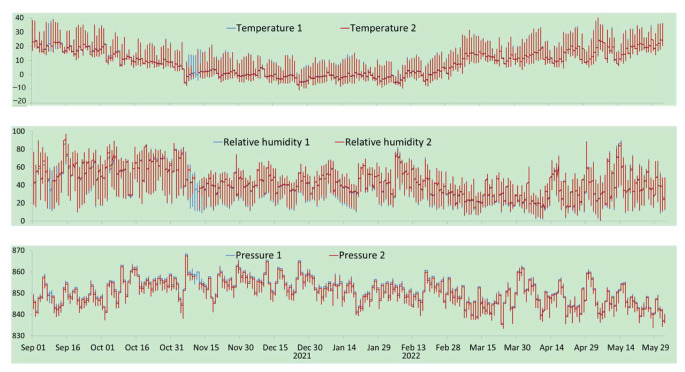
Real-time monitoring data for temperature (°C, **top**), relative humidity (%, **middle**), and pressure (hpa, **bottom**) from the monitoring system from 1 September 2021.

Some data are shown in Figure 9. It can be seen from the figure that the system operates stably and can adapt to conditions for field deployment.

## 5. Conclusions and Outlook

The system has characteristics such as providing wireless, real-time monitoring of the internal parameters of mountain glaciers, and the main advantages are summarized as follows.

Similar to a piece of gravel, a glacier’s internal measurement unit can move with the glacier, and the wireless system can obtain true movement trajectories without the constraints of communication cables. After more than two years of tests and numerous upgrades, the unit now consumes less energy (sleepy mode: the current is only 3 µA), automatically measures real-time data according to the time interval set by the program, and the glacier base station completes the launch, save, and upload functions, including the upload of measurement data to the cloud data platform (OneNET IoT Platform). The data transmission efficiency is high and up to 2.4 kbps. The ultra-low power design in the glacier internal measurement unit permits operations to last within the range of 4 to 6 years. The network is safe for the high integration between the glacier internal measurement unit and the glacier surface base station. Temporary or seasonal field-monitoring network systems can be quickly set up to complete the measurement and upload the related parameter data instead of using manual operations. Without returning to the field to retrieve data, field costs can be dramatically reduced. The system can monitor internal movements, but this is not limited to mountain glaciers. With the improvement in the design scheme, in further research studies, the future system can also be placed in another cryosphere (such as frozen soil) to meet the actual scientific research needs. The system’s design will benefit the measurements of velocity and electrical conductivity at the bottom of a glacier, and it provides valuable technical experiences.

Currently, we are focusing on comprehensive tests for the software and hardware system, and we are summarizing the problems found in the relative tests and improving the design scheme. The next step is to complete the assembly and debugging the first-generation model as soon as possible and to put it into operation in a glacier. If possible, the system will be deployed at the glacier bedrock, since the basal melt is a non-negligible component of the mass balance, and basal melt-water production is likely increasing and will continue to do so in the foreseeable future [47]. We understand that this wireless monitoring system is still in its infancy in comparison to wired sensors, and that there is still much to learn. Nonetheless, this monitoring system would be beneficial for future projections on the movement velocity of the bottom of a glacier. With this demonstrated wireless monitoring system, a powerful tool is now available for quantitatively investigating a glacier’s internal movement, for further understanding the similarities and differences in enhanced warming relative to different glaciers and for offering early warnings of glacial disasters.

## Figures and Tables

**Table 1:** Detailed system power consumption (one day, one unit).

	Supply (V)	Max Current (A)	Sleepy Current (µA)	Consumption 1		Consumption 2	
				Max Power (W)	Last (s)	Sleep Power (W)	Last (s)
Unit	3.6	0.248	3	0.894	34.5	0.00001	83,855
Station	12	0.344	460	4.12	48.9	0.006	86,034.4

Note: (1) Unit refers to the glacier internal measurement unit, and station refers to the glacier surface station. (2) The unit sending phase lasts about 12 min, and the station’s uploading phase lasts about 5 min. Power consumption is determined by the signal at the time of transmission.

## Data Availability

Not applicable.
